# Climate change in the oceans: evolutionary versus phenotypically plastic responses of marine animals and plants

**DOI:** 10.1111/eva.12109

**Published:** 2013-10-14

**Authors:** Thorsten B H Reusch

**Affiliations:** GEOMAR Helmholtz-Centre for Ocean Research Kiel, Marine Ecology – Evolutionary Ecology of Marine FishesKiel, Germany

**Keywords:** adaptation, genetic diversity, ocean acidification, ocean warming, phenotypic buffering, physiological tolerance, selection

## Abstract

I summarize marine studies on plastic versus adaptive responses to global change. Due to the lack of time series, this review focuses largely on the *potential* for adaptive evolution in marine animals and plants. The approaches were mainly synchronic comparisons of phenotypically divergent populations, substituting spatial contrasts in temperature or CO_2_ environments for temporal changes, or in assessments of adaptive genetic diversity within populations for traits important under global change. The available literature is biased towards gastropods, crustaceans, cnidarians and macroalgae. Focal traits were mostly environmental tolerances, which correspond to phenotypic buffering, a plasticity type that maintains a functional phenotype despite external disturbance. Almost all studies address coastal species that are already today exposed to fluctuations in temperature, pH and oxygen levels. Recommendations for future research include (i) initiation and analyses of observational and experimental temporal studies encompassing diverse phenotypic traits (including diapausing cues, dispersal traits, reproductive timing, morphology) (ii) quantification of nongenetic trans-generational effects along with components of additive genetic variance (iii) adaptive changes in microbe–host associations under the holobiont model in response to global change (iv) evolution of plasticity patterns under increasingly fluctuating environments and extreme conditions and (v) joint consideration of demography and evolutionary adaptation in evolutionary rescue approaches.

## Introduction

The ocean is by far the largest habitat on planet Earth. Even larger is our level of scientific ignorance with respect to basic knowledge on its biodiversity. While the recently completed census of marine life compiled a list of 240 000 metazoan species known to science, three to nine times more species still await discovery and description, depending on the extrapolation approach used (Mora et al. 2011). The relationship between known compared with undescribed microbial diversity is even more sobering. Recent estimates suggest that we currently know <0.1% of the diversity in terms of bacterial, archaeal and viral species (Simon and Daniel 2011).

Global climate change in the oceans is already now affecting species’ physiology (Somero 2010) and the distribution (Poloczanska et al. 2013) and composition of communities (Perry et al. 2005). Latitudinal range shifts as response to warming often surpass terrestrial estimates several fold (Jones et al. 2010; Poloczanska et al. 2013), and emerging mismatches in phenologies may ultimately threaten trophic linkage and hence ecosystem functioning (Beaugrand et al. 2003). In contrast to such physiological and ecological effects, evolutionary adaptation to global change only recently received increasing attention in marine systems (but see Pistevos et al. 2011; Sunday et al. 2011; Lohbeck et al. 2012; Dam 2013; Kelly and Hofmann 2013).

The lead article of the current review series (Merilä and Hendry 2013) focuses on changes in phenotypes through time (allochronic studies) and asks whether or not observed changes are due to phenotypic plasticity or evolutionary change. In marine systems, however, for the overwhelming majority of nonvertebrate animals and plants, no data series are available other than abundance and distribution that document phenotypic change in populations, for example in reproductive timing, behaviour, morphology or growth rates. Exceptions are flowering data in the endemic Mediterranean seagrass *Posidonia oceanica* and growth rates in the brown seaweed *Ascophyllum nodosum* that suggest a link between sea surface temperatures and flowering intensity (Keser et al. 2005; Diaz-Almela et al. 2007). Yet, these changes were probably entirely attributable to phenotypic plasticity, while it is unlikely that they have resulted from (and were not interpreted as) adaptive evolution, as *P. oceanica* and *A. nodosum* are both long-lived plants. Only in populations of fishes (see review by Crozier and Hendry 2013) population-level data of maturation ages and growth rates suggest temporal changes partly attributable to adaptive evolution as a result of size-selective harvesting (Jorgensen et al. 2007; Swain et al. 2007).

Hence, this review has to focus on studies that address the *potential* for phenotypic evolution based on indirect approaches. These are mostly synchronous studies comparing populations coming from divergent habitats in space-for-time (=laboratory common garden) or reciprocal transplant approaches. In such studies, the phenotypically plastic component of phenotypic variance is usually not directly estimated, but contained within the error variance. Indirect evidence is also available from assessments of relevant within-population genetic diversity, for example in tolerance traits in the face of warming or ocean acidification stress, which may then be combined with population genetic projections on adaptation rates (Sunday et al. 2011; Kelly et al. 2013). In contrast, direct experimental evidence on evolutionary adaptation is rare and mostly deals with short-generation time phytoplankton species (Lohbeck et al. 2012; Jin et al. 2013), which are covered by a companion review in this issue (Collins 2014). However, there are a few exceptions from marine animals (Kelly et al. 2012), and these experimental evolution approaches hold great promise as they provide direct evidence for *in situ* adaptive evolution to changing environments.

At a first glance, the marine environment may not seem too conducive to adaptive evolution compared with land. One salient difference to terrestrial environments is marine connectivity, potentially connecting all locations/habitats via genetic exchange of adults, larvae, spores or other propagules (Palumbi 1994). This should move the balance between spatially divergent selection on one hand, and gene flow on the other away from adaptive changes as a result of selection (Bolnick and Nosil 2007). However, there are few actual examples in marine species where gene flow prevents or slows down local adaptation. To the contrary, the many examples of local adaptation in marine invertebrates, in particular to temperature regimes, (Helmuth et al. 2006; Sanford and Kelly 2011) suggest that locally divergent selection often overrides homogenizing effects of gene flow (Schmidt et al. 2000). At the same time, it turns out that dispersal is more complex and spatially confined than previous simplistic scenarios have predicted (Levin 2006). Realized dispersal among contrasting habitats may also be drastically reduced by phenotype–environment mismatch of dispersing propagules (Marshall et al. 2010), also called ‘selection against immigrants’ (Hendry 2004).

On the other hand, marine species should possess large standing genetic diversity and hence display a high evolutionary potential. Many marine populations, in particular, species in the plankton as well as mass-spawning ones with numerous planktotrophic larvae, should possess much larger population sizes and hence higher standing genetic diversity compared with species/populations on land. A critical concept was already introduced by Wright (1931), the effective population size *N*_e_, the size of a hypothetical ideal population with random mating that corresponds to population genetic processes within the focal wild population. When the product of the selection coefficient *s* (defining the fitness differential between two alleles) and *N*_e_ is <1, then, random processes (genetic drift) will constrain adaptive responses via selection. Population size has been invoked to be one key variable for the possibility of evolutionary rescue (ER) of populations under changing environments, either by determining the amount of quantitative genetic variation responsive to selection, or indirectly via inbreeding effects (Willi et al. 2006). Unfortunately, there are very few population genetic estimates of effective population sizes (as are estimates of selection coefficients) in marine systems (Hare et al. 2011). Most examples, again, come from fish (Crozier and Hendry 2013), while for most marine invertebrates, only ecological census estimates are available (but see Ovenden et al. 2007; De Wit and Palumbi 2013), which may diverge widely from *N*_e_ (Zeller et al. 2008). The most relevant approach for estimating *N*_e_ is contemporary temporal methods, which operate at the same time scale as the adaptation processes in response to global change (Hare et al. 2011). While many mass-spawning vertebrates (fish) and invertebrates are likely to posses *N*_e_ values that will not constrain selective responses, this may not apply to small populations confined to fringe habitats (for example tide-pool copepods, Kelly et al. 2012) or to large-bodied species such as elasmobranchs (Chevolot et al. 2008), marine mammals (Alter et al. 2007) or large marine plants (Reusch et al. 1999).

## A brief glance on the future ocean

The ocean environment is characterized by strong vertical and horizontal gradients in several abiotic factors, such as light, turbulence, concentrations of dissolved elements, oxygen, hydrostatic pressure and temperature, some of which show diurnal and seasonal fluctuations notably in light levels and temperature. Superimposed onto these existing gradients, a multitude of environmental factors are predicted to change in mean and variances in the coming decade (Boyd et al. 2010). The scope of this review in terms of selection factors is dictated by the available literature, which mostly deals with ocean warming, ocean acidification and deoxygenation. Marked warming trends in surface waters are apparent already today sometimes markedly exceeding atmospheric warming (Perry et al. 2005). Even water layers that are relatively isolated from the well-mixed surface ocean already begin to reveal measurable warming signal down to 1000-m water depth (Roemmich et al. 2012). Due to the large latent heat of water, extreme values are often buffered. On the other hand, once critical temperature thresholds are reached, no microhabitats are available to mobile organisms for escaping, nor is evaporative cooling possible (Bergmann et al. 2010), in contrast to the situation for terrestrial invertebrates (Schilthuizen and Kellermann 2013). In tropical areas, many organisms live close to their upper thermal limit, such that small absolute increases in water temperature of only 1–2°C may result in severe mortality selection, as is the case for species of reef-building (scleractinian) corals (Pandolfi et al. 2011).

Excess carbon dioxide from fossil fuel burning is also directly affecting ocean water chemistry. As a result of direct dissolution of CO_2_, ocean waters become less basic. This process, dubbed ocean acidification, profoundly alters the abundance of different inorganic carbon species and interferes with a range of processes, including growth, calcification, development, reproduction and behaviour (Orr et al. 2005; Kroeker et al. 2010). Importantly, the predicted drop in ocean pH and increase in pCO_2_ are faster and of greater magnitude than any event since the past 300 million years (Caldeira and Wickett 2003). Larger marine animals/ontogenetic stages with large volumes of extracellular space are impacted by elevated pCO_2_, as they need to maintain a positive CO_2_ gradient from the body fluids to the environment to excrete metabolic CO_2_ via diffusion (Melzner et al. 2009). Changes in ocean acidification thus lead to higher body fluid pCO_2_ in animals, which causes acid–base disturbances. These, in turn, can lead to reallocation of resources not available for other functions such as growth and reproduction, which likely translate into impaired fitness. On the other hand, regulatory energy expenditure can be compensated by high resource availability, for example of food to filter feeders (Thomsen et al. 2013). This contrasts to the situation in unicellular organisms and gametes, as well as small ontogenetic stages (larvae) for which the ocean is the extracellular space. Here, physiological tolerances cannot be compensated by energy-expensive regulation that makes these life-history stages/organisms more vulnerable to ocean acidification effects (Melzner et al. 2009).

Calcifying animal and plant species are additionally impacted in their ability to precipitate biogenic carbonate by lowered pH and carbonate ion concentrations (Kroeker et al. 2010). Their sensitivities and hence the intensity of selection imposed by future level of ocean acidification depend on the detailed physiological mechanism. For example, decreased carbonate concentrations have been shown to exert a direct influence on calcification rates of mussel larvae, foraminifera or reef-building corals (Bentov et al. 2009; Gazeau et al. 2011). For other species, the direct pH effects seem to be more important, for example in coccolithophores (Bach et al. 2013). Morphological structures may also matter. For example, in some species of bivalves, the periostracum, an organic shell cover protecting carbonate shells from ocean waters under-saturated with carbonate, may enable biogenic calcification even in corrosive waters as has been shown in deep-sea mussels inhabiting highly acidic hydrothermal vent areas (Tunnicliffe et al. 2009).

As the pCO_2_ in the atmosphere is continuing to rise, this also enhances the availability of inorganic carbon to marine photosynthetic autotrophs such as macroalgae and seagrasses (Harley et al. 2012), phytoplankton (Riebesell and Tortell 2011) and unicellular symbionts associated with metazoan hosts (Zilber-Rosenberg and Rosenberg 2008), with positive effects on plant growth rates, reproduction and photosynthesis. However, as the lower availability of 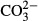 ions along with increased pCO_2_ can impede calcification, photosynthesis and growth of calcifying autotrophs including calcifying macroalgae, reef-building corals and calcifying unicellular plankton are often negatively impacted (reviewed in Kroeker et al. 2010).

Spatial gradients in ocean pH and CO_2_ availability are less well defined than for temperature with the exception of CO_2_ vents (Hall-Spencer et al. 2008; Rodolfo-Metalpa et al. 2011) and CO_2_-enriched coastal habitats (Feely et al. 2008) where natural high pCO_2_ habitats can be contrasted to surrounding area with ambient CO_2_ values. This opportunity has not yet been explored except in one recent study (Kelly et al. 2013).

An environmental change interacting with warming and stratification that will become more severe in the coming decades is hypoxic (oxygen-poor) periods or entire regions in both open ocean and coastal areas (Diaz and Rosenberg 2008). Hypoxic zones, in turn, are always correlated with locally high pCO_2_ values and low carbonate concentrations due to excess respiration (Feely et al. 2008). Hence, at the same time, they may provide test cases for ocean acidification status today that otherwise is predicted for the next century in more oxygen-rich areas (Feely et al. 2008; Melzner et al. 2013).

## Modes of evolution and selection in the brave new ocean

An important issue to understand adaptive responses is the nature of selection via global change (Franks and Hoffmann 2012). One the one hand, the key variables of the present review, ocean acidification and warming, may have immediate beneficial (i.e. fitness-enhancing) effects. For example, increased availability of inorganic carbon (as dissolved CO_2_) will enhance the growth of marine plants (Harley et al. 2012). Elevated mean ocean temperatures may mean longer growth periods, a favourable condition that benefits those genotypes that can readily take advantage by enhancing their reproduction and growth rates (Dehnel 1955; Eggert et al. 2005). Summer heat waves, on the other hand, may constitute sublethal stress in seagrasses (Reusch et al. 2005), corals (Howells et al. 2011), gorgonians (Cerrano et al. 2000) and marine invertebrates (Moore et al. 2011).

**Figure 1:**
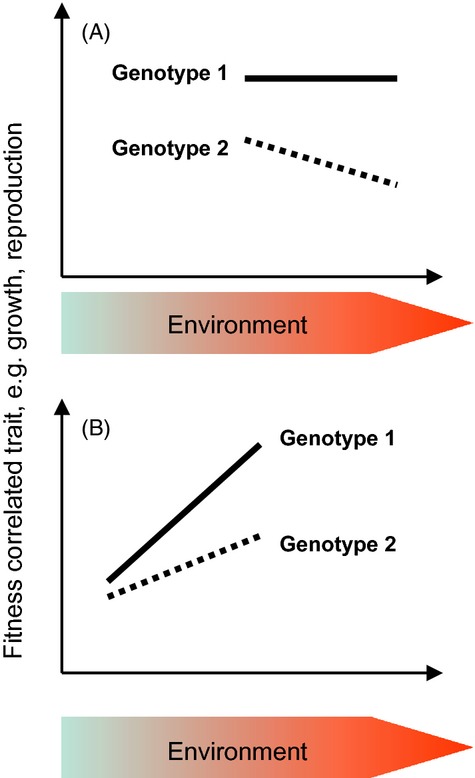
Hypothetical reaction norms depicting a fitness-correlated trait such as growth or reproduction as function of a changing environmental variable (e.g. temperature, CO_2_ availability). The variable can represent a stressor (A) or represent enhanced opportunity (B), depending on the physiology of the species, and the magnitude of the factor. The genotype with the solid line will be favoured by selection. In (A), genotype 1 is maintaining its function, thus shows better *phenotypic buffering* than genotype 2. The corresponding reaction norm is flat. In (B), genotype 1 is more *phenotypically plastic*; thus, the slope of the reaction norm is steeper than of less plastic genotype 2. Here, selection would favour genotype 1 over 2 as the former can readily take advantage of the improved environmental condition. See Box 1 for more details.

In the case of selection for increased opportunity, those genotypes that possess more plasticity, *sensu* a steeper slope of the reaction norm with increasing inorganic carbon availability, will profit more, and when the shape of the reaction norm is heritable, adaptive evolution will take place, here in the form of lineage sorting of preadapted genotypes (Schaum et al. 2013; Fig. 1B). There are also recent theoretical advances that predict faster evolutionary rates and higher likelihood of population persistence if plasticity itself can evolve (i.e. the slope of the reaction norm), but this only applies to selection for opportunity (Chevin et al. 2013a,b) and not to phenotypic buffering (Box 1, Fig. 1).

**Box 1: Phenotypic plasticity versus phenotypic buffering**

Phenotypic plasticity broadly defines the adjustment of phenotypic values of genotypes depending on the environment, without genetic changes. Originally, it describes *different* phenotypes produced by the same genotype as a function of the environment (Schlichting and Pigliucci 1996). Difficulties arise with this definition when dealing with traits closely correlated with fitness such as growth, reproduction and mortality in stressful environments. The (adaptive) maintenance of a functional phenotype in the face of environmental stress essentially translates to the *same* phenotype produced by an underlying genotype. Confusion arises when such a genotype is described as being ‘more plastic’. When depicting the reaction norms (i.e. average trait value of a genotype versus environment) (Fig. 1, see also Box 1 in Pigliucci 2005), the reaction norm would essentially be a flat line in a genotype with appropriate tolerance traits (Schlichting and Pigliucci 1996). The latter case should rather be termed phenotypic buffering, a special case of plasticity (Waddington 1942; Bradshaw 1965). In contrast, reaction norms with a nonzero slope in response to the environment describe phenotypic plasticity of traits *sensu stricto*. Some authors therefore distinguish tolerance curves, depicting tightly fitness-correlated traits such as growth and survival, from reaction norms that describe traits with a more complicated connection to fitness (Chevin et al. 2010).

The two plasticity types are associated with different modes of selection by global change. Classical plasticity is most relevant under selection for enhanced opportunity (Franks and Hoffmann 2012), here those genotypes are favoured that can adaptively adjust their phenotype to rapidly take advantage of novel conditions, such as earlier hatching for a seasonal insect as a result of increased mean temperatures (Bradshaw and Holzapfel 2001), or more dissolved inorganic carbon for microalgae (Schaum et al. 2013). In contrast, when the environmental change translates to enhanced stress levels at the edge of tolerance ranges, selection is for enhanced tolerance, that is, phenotypic buffering. Note that it is likely that other levels of biological organization need to respond in a truly plastic way to accommodate external stress and maintain homeostasis (Schlichting and Pigliucci 1995). Key examples are the increased expression of shock proteins to maintain proper cellular metabolism as a response to heat stress (Sorensen et al. 2003; Bergmann et al. 2010; Csaszar et al. 2010).

Phenotypic buffering is by definition adaptive when it confers the maintenance of organismal functioning. Only when buffering collapses, nonadaptive alternative phenotypes may be expressed, largely as a consequence of stress (Fig. 1A, genotype 2). For selection under enhanced opportunity, the fitness advantage of the more plastic genotype depicting a steeper slope (Fig. 1B, genotype 1) needs to be formally demonstrated. If plasticity itself can evolve, here the slope of the reaction norm in a linear model, then plasticity will help maintaining populations under changing environments (Chevin et al. 2010; Chevin et al. 2013a,b). An interesting (and unresolved) question is whether or not global change will impose selection directly upon plasticity, in particular, when environmental variance rather than mean values increase (Thompson 1991; Pigliucci 2005; Chevin et al. 2013a,b).

Moreover, increased duration of the growth period under increased mean temperatures may turn current patterns of countergradient variation (Conover and Present 1990) that maintain nearly constant life-history traits in latitudinally distributed species become maladaptive (Bradshaw and Holzapfel 2001). For example, it may become beneficial to produce less diapausing versus direct developing eggs under warmer climates. If latitudinal variation exists for developmental modes and diapausing cues, adaptive evolution of local populations to accommodate enhanced opportunities due to ocean warming may take place, as has been shown for coastal copepod species (Marcus 1984; Avery 2005). These are the only studies that suggest the possibility of adaptive evolution of life-history cues in the marine realm (see terrestrial examples in companion reviews by Franks et al. 2013; Charmantier and Gienapp 2013), rather than first-order effects on organismal physiology.

A fundamentally different selection regime is in the face of increasing stress. Here, selection is in favour of genotypes displaying increased tolerances that are thus able to maintain organismal function despite environmental deterioration. This process needs to be distinguished from phenotypic plasticity in its original meaning and has been dubbed phenotypic buffering before (Waddington 1942; Bradshaw 1965; Box 1, Fig. 1). In the context of tolerance selection, the precise pattern of duration and intensity of stress is as important as are elevated mean values, as is the case for selection for enhanced opportunity.

## Potential for adaptive evolution – the evidence in marine systems

Our knowledge from marine systems is fragmentary and encompasses very few studies that follow populations over time with phenotypic data other than abundance and distribution. The few exceptions either deal with long-lived organisms that preclude adaptive responses (Keser et al. 2005; Diaz-Almela et al. 2007) or do not provide any evidence for adaptive components of the phenotype (Moore et al. 2011). The best examples for an evaluation of plastic versus adaptive changes are probably from the fish world (see companion review by Crozier and Hendry 2013). Here, individual-based measures of maturation reaction norms, reproductive investment and growth rates provide compelling evidence for evolutionary change due to harvesting (Olsen et al. 2004; Swain et al. 2007). There are also no studies where individual traits are repeatedly measured throughout generations or related individuals, which precludes any animal model approaches laid out by Merilä and Hendry (2013). This is in contrast to terrestrial species where, for example, flowering time, migration patterns, dispersal traits, behaviour or reproductive timing have changed as phenotypic or genetic response to more favourable climatic conditions (references to be added from other reviews, this issue).

Another way to demonstrate evolutionary adaptation is the direct assessment of genetic changes within the genomes of the focal populations. Yet, I am unaware of any successful association of *causal* genetic change at the DNA level that links observed phenotypic change to its genetic basis in marine systems. This lack is not a general shortcoming of marine studies, but reflects the general difficulty to associate the genotype with a phenotype for most but the simplest traits and adaptations (Travisano and Shaw 2013). However, some molecular phenotypes, in particular gene expression patterns, are consistent with physiological divergent phenotypes, for example in terms of thermal adaptation and tolerance (Somero 2010; Franssen et al. 2011). Here, it was often observed that population-specific patterns in expression of heat shock protein genes (*hsps*) are broadly consistent with the thermal niche of a population, that is, individuals from colder locations indicated heat stress at lower temperatures by expressing *hsp* genes in marine invertebrates and seagrasses (Osovitz and Hofmann 2005; Bergmann et al. 2010). Other evidence for divergent selection operating at the molecular genetic level come from comparisons of enzyme DNA sequence (Somero 2012) and from genome scans. Recent examples include populations of red abalone (De Wit and Palumbi 2013) and purple sea urchins (*Strongylocentrotus purpuratus)* (Pespeni et al. 2013) that came from different thermal or ocean acidification habitats, respectively (De Wit and Palumbi 2013; Pespeni et al. 2013). In the study by Pespeni et al. (2013), a temporal genome scan upon exposure of sea urchin larvae revealed dozens of alleles that changed relative to control CO_2_ conditions. An excess of nonsynonymous over synonymous nucleotide substitutions in CO_2_-favoured alleles corroborated the hypothesis that acidification-induced selection was responsible for population genetic changes. It is noteworthy, however, that there were no detectable phenotypic differences among the urchin families of different parental origin.

In marine systems, the bulk of evidence addressing the potential of adaptive evolution versus plastic responses come from two approaches. In synchronic comparisons of populations, many case studies report phenotypic differences that are consistent with local adaptation among subpopulations from contrasting habitats (reviewed for coastal animals in Helmuth et al. 2006 and Sanford and Kelly 2011). Second, an increasing number of studies using breeding designs/comparisons of clonal genotypes identified (additive) genetic variance in traits such as CO_2_ or temperature tolerance. As such approaches only describe the potential for adaptive evolution, this approach was not explicitly included in the lead review (Merilä and Hendry 2013). The focal traits were predominantly physiological responses and tolerances, thus ‘labile’ traits that can be adjusted several times during the life time of an organism. Few studies addressed life-history cues, for example for diapausing, while I am not aware of a single study addressing developmental traits that can only be adjusted once during ontogeny, in contrast to many terrestrial studies (Franks et al. 2013; Charmantier and Gienapp 2013; Schilthuizen and Kellermann 2013).

## Evidence from synchronic approaches

In synchronic approaches, the end result of past evolutionary adaptation can be tested using two designs. In laboratory experiments, individuals from divergent locations that are putatively locally adapted with respect to a hypothesized factor such as temperature regime or pCO_2_ are exposed to different levels of that factor in the laboratory (common garden approach), ideally under at least two levels of that factor to unravel G × E (genotype × environment) interactions (Falconer and McKay 1998). A second approach is reciprocal transplant experiments. Here, adaptation to local conditions is visible though better performance of local versus foreign genotypes (Kawecki and Ebert 2004), but any interpretation is difficult owing to the multivariate nature of diverging habitats. A possible solution is to use multiple environmental contrasts with respect to the focal factor, say temperature, to remove idiosyncratic effects of specific localities (Kawecki and Ebert 2004).

### Brief overview on available evidence – plants

In marine plants (excluding phytoplankton), adaptive phenotypic divergence at the population level was mainly studied with respect to temperature regimes (Table 2), either in common garden or in reciprocal transplant designs. For macroalgae and seagrasses, global change constitutes a complex mixture of immediate positive and negative effects. For noncalcifying seaweeds and seagrasses, the increased availability of inorganic carbon through dissolution of CO_2_ in ocean waters alleviates nutrient (inorganic carbon) limitation and enhances growth (Harley et al. 2012). This does not apply to many calcifying algae that have difficulties to produce calcium carbonate under increasing acidification (Kroeker et al. 2013). Thus, in the former case, selection is for enhanced opportunity, while tolerance evolution is relevant to calcifying forms to compensate for higher costs of calcification under lower carbonate saturation. To the best of our knowledge, there are no temporal or spatial studies addressing adaptation to ocean acidification in any macroalgae or seagrass, neither for tolerance nor for enhanced opportunity.

Increasing mean temperature predicted for many regions will strongly interact with genetically based seasonality patterns that are probably highly adaptive both within and among species. Warmer waters may enable local algal populations to grow longer time periods when conditions become more favourable, typically at colder sections of their current distribution range (Eggert 2012). However, when populations grow at the upper end of their thermal tolerance, which applies particularly to tropical species, adaptation may occur in response to increasing stress. In many geographically widespread macroalgae, the presence of thermal ecotypes suggests that local adaptation to the prevalent temperature regime is possible (Breeman 1988; reviewed in Eggert 2012). As many algae have complicated two- or three-phasic life cycles, predicting the adaptive responses and associated selection regime requires the inclusion of the full life cycle, which has seldom been done (Harley et al. 2012).

For the dominant seagrass of the Northern Hemisphere, *Zostera marina* (eelgrass), a series of common garden experiments have revealed some evidence for thermal adaptation of southern versus northern populations in terms of their photophysiology (Winters et al. 2011). At the same time, transcriptomic resilience, the recovery to normal gene expression patterns, was consistent with the observed temperature tolerance in southern populations under a simulated summer heat wave (Franssen et al. 2011), while northern populations were lacking such resilience. Such transcription patterns may be one important correlate to address phenotypic buffering at the molecular genetic scale.

### Animals

In marine animals, synchronic approaches focus on divergent thermal ecotypes, with most studies coming from cnidarians (reef-building corals), gastropod molluscs and copepods (Crustacea). Most studies used controlled laboratory common garden designs under space-for-time substitution approach, manipulating either a range of temperatures including stressful values, or only temperature as stressor (Table 2). About half of the published evidence deals only with tolerances at the upper end of the range of temperatures, while half addresses both selection for enhanced opportunity and tolerance (Table 1). Some studies addressed correlated responses other than tolerances that place the first-order physiological response into an ecological context. For example, in the intertidal copepod *Tigriopus californicus,* Willet (2010) found that the competitive fitness of genotypes from different thermal habitats differed in a way consistent under a thermal adaptation hypothesis, that is, warm-adapted individuals displaced cold-adapted ones under high temperature stress.

**Table 1:** Glossary for terms used in this review

Term	Explanation
Coral bleaching	Loss of dinoflagellates (genus *Symbiodinium*), endosymbiotic unicellular algae from reef-building corals as response to thermal or other stress
Calcification	Biogenic production of calcium carbonate in the form of shells, scales, spicules or skeletons in marine animals and plants
Corals	Reef-building (scleractinian) corals are cnidarians and form long-lived colonies that may construct reefs of hundreds of km in dimension
Counter-gradient variation	Variation in the reaction norm of a phenotypic trait that compensates for a gradient for example in temperature, maintaining for example development time or body size across latitudes
Genetic assimilation	Population genetic process coined by Waddington describing how a phenotypically plastic trait becomes subsequently genetically fixed within the extreme range of environments
Holobiont	Host organism (animal and plant) along with its entire diversity of associated prokaryotic and eukaryotic-associated microbes
Macroalgae	Multicellular photoautotrophic protists that are of diverse phylogenetic origin, important members are red algae, brown algae (e.g. kelps) and green algae. The latter gave rise to higher land plants
Metapopulation	Network of subpopulations connected via dispersal, characterized by extinction and recolonization processes
Ocean acidification	Decrease in ocean pH due to the dissolution of anthropogenic (excess) carbon dioxide derived from fossil fuel burning
Phenotypic buffering	Maintenance of a functional phenotype under stressful conditions, that is, to tolerate bad environmental conditions, applies mostly to tightly fitness-correlated traits such as growth and reproduction
Phytoplankton	Microscopically small autotrophic unicellular ‘plants’ of very diverse phylogenetic origin that contribute to the bulk of primary productivity in the ocean
Planktotrophic	Nutritional type of many larvae of marine invertebrates that feed on plant and animal plankton during the first days to weeks until they metamorphose and settle to the seafloor
Seagrasses	Polyphyletic group of flowering plants that returned secondarily to the marine habitat
Selection for opportunity	Selection regime under global change when changing conditions represent more favourable conditions that could be exploited if traits such as maximal growth rates evolve
Symbiont	Unicellular protists and prokaryotes closely associated with metazoan host organisms, their role can be beneficial, neutral or pathogenic

Only two studies addressed the population-level differentiation in traits related to seasonality. In a controlled laboratory study using the F1 generation of a copepod species (*Labidocera aestiva*), the production of dormant eggs was population specific, suggesting local adaptation of developmental mode to the length of the growing season, which is covarying with temperature (Marcus 1984). Another seasonal adaptation, summer dormancy, was found to vary among populations in another copepod species, *Acartia hudsonica* (Avery 2005). Both these studies highlight that population-level phenology and life-history transitions vary within populations and may undergo adaptive evolution with altered temperature regimes, similar to patterns observed on land (Bradshaw and Holzapfel 2006; Schilthuizen and Kellermann 2013). One interesting study with respect to oxygen deficiency as stress selection is available in the coastal copepod species *A. tonsa*. Here, population-level differences were found with respect to behavioural avoidance of hypoxia only in those populations that came from an estuary often suffering from low oxygen (Dekker et al. 2003).

There are far fewer studies addressing adaptation to ocean acidification using a synchronic approach. Using the well-defined CO_2_ gradient of the Ischia vent site, calcification rates of limpets coming from low and control pH sites were examined under controlled high and low pH conditions. Limpets from close to the vent calcified more under all conditions, suggesting some adaptively increased calcification rates (Rodolfo-Metalpa et al. 2011). However, it is unclear whether this is a true genetically based adaptation, or whether this represents long-term acclimation (e.g. Dupont et al. 2013). Recently, Kelly et al. (2013) bred sea urchin larvae (*Strongylocentrotus purpuratus*) from populations diverging in the pH environment their parents experience along the Pacific coastline, owing to different upwelling regimes along with oxygen deficiency and naturally occurring pH drops. The maintenance of larval size was related to experimental ocean acidification stress in a way consistent with local adaptation to naturally occurring pH value decreases.

In synchronic approaches, it is mandatory to erase environmental effects that persist within or even across generations to correctly infer evolutionary adaptation. Unfortunately, even long-term acclimation within generations may be insufficient to erase irreversible environmental effects. For example, early ontogenetic effects on muscle morphology and swimming performance in zebrafish were found to be unaffected by subsequent acclimation of adults to different thermal regimes (Scott and Johnston 2012). Likewise, early-phase exposure of juvenile oysters to OA persisted to the juvenile stage regardless of later treatments (Hettinger et al. 2012). Even more sobering are recent findings on trans-generational carry-over effects in a range of marine invertebrates exposed to ocean acidification (Parker et al. 2012; Dupont et al. 2013) or in fish species exposed to warming, ocean acidification and hypoxia (Donelson et al. 2011; Miller et al. 2012; Salinas and Munch 2012). Thus, for most studies, we cannot exclude the possibility that long-term carry-over effects including epigenetic inheritance can influence estimates of trait value divergence obtained, although the assay conditions were properly controlled. An ideal design would be to propagate populations within the laboratory for at least two generations, which was only realized in 5/23 studies compiled in Table 2. However, even breeding until the F2 generation may not be sufficient to control for trans-generational carry-over effects (Schmitz and Ecker 2012).

**Table 2:** Synchronic studies in marine systems demonstrating past local adaptation to global change-associated environmental parameters. Plasticity components to the phenotype were not separately estimated

Taxonomic affiliation	Species	Trait type	Genetic	Cause	Primary driver	Reference
Plant studies						
Chlorophyta	*Valonia utricularis*	GR, SV	5 (F > 10)	1	T (R + S)	Eggert et al. (2005)
Chlorophyta, Rhodophyta, Phaeophyta	18 species of macroalgae	GR, SV	5 (F > 10)	1	T (R + S)	Breeman and Pakker (1994)
Planta, Spermatophyta	*Zostera marina*	PS	5 (FC)	1,2	T (S)	Winters et al. (2011)
Animal studies						
Cnidaria, Anthozoa	*Metridium senile*	MR, EA	5 (FC)	1,2	T (S)	Walsh and Somero (1981)
Cnidaria, Hexacorallia	*Pocillopora damicornis*	O*/†	5 (FC)	1,2	T (S)	D'Croz and Mate (2004)
Cnidaria, Hexacorallia	*Pocillopora damicornis*	O*/†, PR	5 (FC)	1,2	T (S)	Ulstrup et al. (2006)
Cnidaria, Hexacorallia	*Turbinaria reniformis*	O*/†, PR	5 (FC)	1,2	T (S)	Ulstrup et al. (2006)
Crustacea, copepoda	*Acartia hudsoncia*	DP	5 (F2)	1,2	SE	Avery (2005)
Crustacea, copepoda	*Labidocera aestiva*	DE	5 (F1, F2)	1,2	SE	Marcus (1984)
Crustacea, copepoda	*Tigriopsis californicus*	SV, CO	5 (F2…F5)	1,2	T (S)	Willet (2010)
Crustacea, copepoda	*Scottolana canadensis*	GR, SV	5 (F2…F5)	1,2	T (R + S)	Lonsdale and Levinton (1985)
Crustacea, Cirripedia	*Semibalanus balanoides*	SV	7 (FC)	1	T (S)	Bertness and Gaines (1993)
Crustacea, Decapoda	*Uca pugnax*	GR	5 (F1)	1,2	T (R)	Sanford et al. (2006)
Mollusca, Gastropoda	*Crepidula fornicata, C. convexa*	GR	5 (FC, F1)	1,2	T (R + S)	Ament (1979)
Mollusca, Gastropoda	*Crepidula nummaria*	GR	5 (F1)	1,2	T (R + S)	Dehnel (1955)
Mollusca, Gastropoda	*Lacuna carinata*	GR	5 (F1)	1,2	T (R + S)	Dehnel (1955)
Mollusca, Gastropoda	*Lacuna vincta*	GR	5 (F1)	1,2	T (R + S)	Dehnel (1955)
Mollusca, Gastropoda	*Thais emarginata*	GR	5 (F1)	1,2	T (R + S)	Dehnel (1955)
Mollusca, Gastropoda	*Nucella canaliculata*	SV	5 (F2)	1	T (S)	Kuo and Sanford (2009)
Mollusca, Gastropoda	*Nucella emarginata*	GR	5 (F2)	1,2	T (R)	Palmer (1994)
Mollusca, Gastropoda	*Bembicium vittatum*	GR, ‡	5 (F1)	1	T (R)	Parsons (1997)
Echinodermata, Echinoida	*Strongylocentrotus purpuratus*	GR, MR, ‡	5 (F1)	1,2	OA (S)	Kelly et al. (2013)
Echinodermata, Echinoida	*Strongylocentrotus purpuratus*	GE	5 (FC)	1	T (S)	Osovitz and Hofmann (2005)

Trait type: GR, growth rates, SV, survival, PS, photosynthesis, MR, metabolic rates, DP, diapausing time, EA, enzyme activities, CO, competitive ability, GE, gene expression, O, other (see footnote). Genetic evidence: 1, animal model, 2, common garden studies, 3, comparison to model predictions, 4, experimental evolution, 5, space-for-time, 6, molecular genetic evidence, 7, reciprocal transplant. Qualifier for categories 2 and 5: WC, wild collected material, F_x_, use of laboratory-raised progeny of generation x. Cause categories: 1, common sense, 2, experimental (temporal correlation not assessed). Selective driver: T, temperature, OA, ocean acidification, LO, low oxygen, SE, seasonality, qualifier in brackets: R, range of conditions, S, only stressful conditions.

* Zooxanthellae abundance.

† Coral bleaching.

‡ Morphology.

## Assessing within population adaptive genetic diversity

The second line of evidence for the potential of adaptive evolution comes from an assessment of additive genetic variance within focal populations through breeding designs (Table 3) to address the potential for adaptive responses to temperature and ocean acidification. A particularly instructive study dealt with the additive genetic variance in sensitive sea urchin and mussel larvae to ocean acidification (Sunday et al. 2011). Although the sea urchin *Strongylocentrotus franciscanus* has a longer generation time, a population genetic model predicted faster rates of adaptive evolution in sea urchins compared with mussels (*Mytilus trossolus*) because larvae of the latter possessed lower levels of additive genetic variance. The above study only addressed very early larval stages and needs to be extended to later life stages. Other such recent examples include the variation in larval tolerance in a sea urchin to the combined effects of warming and ocean acidification (Foo et al. 2012) and the settlement success of coral larvae in the face of sublethal warming (Meyer et al. 2009). In all cases, significant within-population diversity for the focal traits, here tolerance levels were detected, suggesting the potential for adaptive evolution. Ideally, such studies employ a breeding design that decomposes nongenetic, trans-generational effects from breeding values of genotypes (as in Sunday et al. 2011).

**Table 3:** Population-level studies in marine animals and plants that quantify adaptive genetic diversity with respect to temperature or ocean acidification tolerance

Taxonomic affiliation	Species	Trait type	Genetic	Heritability	Primary driver	Reference
Planta, Spermatophyta	*Zostera marina*	GR, SV	2 (FC)	1	T (S)	Reusch et al. (2005); Ehlers et al. (2008)
Cnidaria, Hexacoralia	*Acropora millepora*	GR, PS, GE	2 (FC)	2	T (S)	Csaszar et al. (2010)
Cnidaria, Hexacoralia	*Acropora millepora*	GR, MR, LS, GE	2 (F1)	1	T (S)	Meyer et al. (2009)
Mollusca, Bivalvia	*Mytilus trossolus*	GR, MR	(F1)	3	OA (S)	Sunday et al. (2011)
Crustacea, Decapoda	*Petrolisthes cinctipes*	MR	(F1)	1	OA (S)	Carter et al. (2013); Ceballos-Osuna et al. (2013)
Echinodermata, Echinoida	*Strongylocentrotus franciscanus*	GR, MR	2,3 (F1)	3	OA (S)	Sunday et al. (2011)
Echinodermata, Echinoida	*Strongylocentrotus purpuratus*	GR, MR, SV	2, 3 (F1)	3	OA (S)	Kelly et al. (2013)
Echinodermata, Echinoida	*Centrostephanus rodgersii*	GR, SV	2 (F1)	1	OA + T (S)	Foo et al. (2012)
Bryozoa	*Celleporella hyalina*	GR	2 (FC)	1	OA + T (S)	Pistevos et al. (2011)

Trait type: GR, growth rates, SV, survival, PS, photosynthesis, MR, metabolic rates, LS, larval settlement, GE, gene expression. Genetic evidence: 1, animal model; 2, common garden studies; 3, comparison to model predictions. Qualifier for categories 2 WC, wild collected material, F_x_, use of laboratory-raised progeny of generation x. Heritability estimate: 1, GxE interaction; 2, broad-sense heritability *H*^2^; 3, narrow-sense heritability *h*^2^. Selective driver: T, temperature, OA, ocean acidification, qualifier in brackets: R, range of conditions, S, only stressful conditions.

Regarding the experimental design, special cases are asexually reproducing animals and plants. Their shoots, runners, branches or subcolonies (=ramets *sensu* Jackson et al. 1985)) allow for a replication of identical genetic material (barring somatic mutations), which makes a comparison of tolerances and associated reaction norms straightforward. For example, in the bryozoan *Celleporella hyalina,* Pistevos et al. (2011) found differences in the tolerance to temperature and OA in terms of growth and reproduction. In a reef-building coral, variation for thermal tolerance was observed both for the host and the symbiont components (Csaszar et al. 2010). In an ecosystem-engineering plant, the seagrass *Zostera marina*, marked among-genotype variation in survival during a heat-stress event was found in the field (Reusch et al. 2005). Interestingly, physiological responses in monoculture with a single genotype differed from the response under competition with other genotypes, suggesting trade-offs between tolerance and competitive ability. Note that in asexually propagated genotypes, among-genotype differences will only provide estimates on broad-sense heritabilities, including an unknown fraction of nonadditive (e.g. epistatic) genetic variance is unknown (Falconer and McKay 1998). Moreover, the risk for substantial nongenetic carry-over effects that inflate heritability estimates is probably high (see above).

The photoperiodic cues to initiate certain life-history phases may be under adaptive evolution (Bradshaw and Holzapfel 2001). For example, the delayed production of dormant eggs for a seasonal diapause is a trait that may to warming waters and associated longer growth periods. In laboratory breeding experiments, it was found that summer dormancy in the copepod species *Acartia hudsonica* has a large heritable component within populations and that the fraction of individuals undergoing summer diapause as a function of day length varies across two populations (Avery 2005). This suggests that an adjustment of the photoperiodic response in northern populations to warming waters via *in situ* local adaptation should in principle be possible.

## Evidence from temporal approaches (experimental and nonexperimental)

To the best of my knowledge, there are no studies in marine systems that track phenotypic traits through time for >10 generations, permitting the detection of temporal changes. In reef-building corals, there are observations that suggest enhanced thermal tolerance after past temperature extremes. These led to massive die-offs (‘coral bleaching’) in many areas of the world (Rowan 2004; Berkelmans and van Oppen 2006). The surviving corals harboured different coral symbiont communities compared with controls. Unicellular algal symbionts are hypothesized to mediate the thermal tolerance, which has also recently been experimentally tested (Berkelmans and van Oppen 2006) and relates to the holobiont concept of (adaptive) evolution (Zilber-Rosenberg and Rosenberg 2008), further discussed below.

Among marine animals and plants, there are very few multigenerational experimental approaches that explore the potential of populations to genetically adapt to global change. One exception is a study on tide-pool copepods along the thermal cline of the East Pacific where possible adaptive responses to warming, including tolerance to temperature extremes, were investigated. Populations of *Tigriopsis* spp. from northern locations failed to adapt to temperature stress in 10 generations of adaptation. Note that *Tigriopsis* is a rather atypical marine invertebrate occurring in exceptionally small, isolated populations. Hence, one likely explanation for the observed evolutionary constraint is the lack of standing genetic variation as a consequence of small *N*_e_ and associated genetic drift (Kelly et al. 2012), which is rare in a marine animal. Another experimental study exposed the pelagic coastal copepod *Tisbe battagliai* over three generations to ocean acidification (Fitzer et al. 2012). In this study, however, the gradual decline of reproductive rates compared with controls allow no inference on adaptation, as no reciprocal exposure experiment was performed that compared control versus OA selection lines under fully crossed conditions (e.g. Collins 2011).

## Adaptive evolution in microbe–host associations

A relatively new finding is that many terrestrial and marine animal and plant species host hundreds of prokaryote and eukaryote microbial symbionts with mostly unknown functional roles along the continuum from mutualism to commensalism to parasitism. Their composition is often markedly divergent from the surrounding environment, while the community composition is often kept relatively stable from generation to generation by a variety of mechanisms (Zilber-Rosenberg and Rosenberg 2008; Wernegreen 2012). The best-studied example is probably the symbiosis between unicellular dinoflagellates of the genus *Symbiodinium* and scleractinian (=reef-building) corals, where *Symbiodinium* photosynthesis provides the host–symbiont association with >90% of its nutrition. Many more examples are appearing in other invertebrates and plants, such as in sponges (Webster et al. 2009), molluscs (Leggat et al. 2000), ascidians (Münchhoff et al. 2007), seagrasses (Bockelmann et al. 2012) and red algae (Harder et al. 2012), to name but a few examples. Under the holobiont model of evolution (Zilber-Rosenberg and Rosenberg 2008), not only the host genotype but also the genotypes of their symbionts contribute to phenotypic variation available to selection (Csaszar et al. 2010). Note that the genetic diversity contained in the microbial symbionts often surpasses that of the associated host several fold (Zilber-Rosenberg and Rosenberg 2008).

There are three mechanisms through which the microbial gene pool may confer adaptation to the holobiont (Zilber-Rosenberg and Rosenberg 2008), (i) changes in microbial composition by differential proliferation within a host (ii) changes in microbial composition by acquisition of new symbiont types from outside and (iii) adaptation of microbial populations of the same species within hosts. Field observations have revealed that upon coral bleaching in response to heat stress, the relative composition of the symbiont community changes among some coral species, with associated increases in thermal tolerance of the holobiont (Rowan 2004; Jones et al. 2008). The causal role for symbiont types on thermal tolerance has recently been demonstrated experimentally (Mieog et al. 2009). Recent findings also suggest that different *Symbiodinium* species have different sensitivities to ocean acidification in experiments with free-living cultures (Brading et al. 2011). In nature, the mechanisms for symbiont community change are both differential replication of standing diversity within hosts (Berkelmans and van Oppen 2006; Silverstein et al. 2012) and possibly, the acquisition of new symbiont types from the environment. Recently, it has also been documented that within one *Symbiodinium* type, adaptive evolution within hosts is in principle possible, as demonstrated by local adaptation to thermal regimes in symbiont populations (Howells et al. 2011), although we do not know the time frame over which such adaptation has happened.

Changes of associated microbes as a response to global change-associated stress have also been reported from several plant and animal species other than reef-building corals (Webster et al. 2008, 2011a,b; Campbell et al. 2011), and there is some evidence that a stable microbial symbiont community assures thermal tolerance to the metazoan host (Webster et al. 2011a,b). There is hence an enormous research gap addressing the role of many other associations among microbes and marine invertebrates/plants under increasing global change induced stress. It is likely that associated microbes have an accelerating role for adaptation, owing to their diversity and fast generation time (Zilber-Rosenberg and Rosenberg 2008; Howells et al. 2011). On the other hand, there are recent reports from terrestrial insects that symbiotic bacteria may constrain thermal adaptation (Wernegreen 2012).

## Nongenetic carry-over effects and global change

In addition to the inheritance mode of the neo-Darwinian modern synthesis, namely information encoded on the DNA (Pigliucci and Müller 2010), additional modes of hereditary transmission of phenotypic traits such as tolerances are highly relevant under rapid environmental change. Such maternal effects can be conceptionalized as trans-generational plasticity or phenotypic buffering, respectively. In marine systems, evidence for a potentially large role of trans-generational plastic effects in response to major drivers of global change, namely ocean acidification and warming, is accumulating. For example, the rate of adaptation to temperature was about 10 times faster via trans-generational plasticity, as opposed to evolutionary adaptation, in a tropical fish (Salinas and Munch 2012). In green sea urchins, the exposure of the parental generation to moderate levels of ocean acidification enhanced the tolerance of larval sea urchins (Dupont et al. 2013). Trans-generational nongenetic effects in response to ocean acidification were studied in Pacific rock oysters (Parker et al. 2012). Here, exposure of adults to elevated pCO_2_ of end-of-the century levels enhanced growth and survival of larvae compared with offspring from parents kept at ambient pCO_2_. This applied to both conditions under which larvae were assessed, CO_2_ exposure and ambient conditions. Strong maternal and nongenetic effects were also reported in the study by Sunday et al. (2011) on within-population genetic variance for ocean acidification tolerance of invertebrate larvae. Here, the dam component of larval size under ocean acidification in urchin and mussel larvae was several fold higher than the narrow-sense heritability.

The non-DNA-based transfer of information from generation to generation can be surprisingly persistent across several generations (Schmitz and Ecker 2012), which means that working with F1 or F2 generations in synchronic approaches may not be sufficient to exclude those. The possible mechanisms are often unresolved, but may include chromatin modification, DNA methylation and the action of small regulatory RNAs (Bossdorf et al. 2008). Epigenetic processes are not mutually exclusive to DNA-based inheritance, but may initially buffer phenotypes and populations in the face of new environmental challenges before genetic assimilation of altered phenotypes (Waddington 1942). There is thus a clear need to decompose the phenotypic responses of marine species into three components, trans-generational plasticity, phenotypic buffering or plasticity within generations, and ‘true’ evolutionary adaptation via DNA-based changes.

## A comparative evaluation of approaches

Among marine animals and plants, most of the available evidence for the potential of adaptive responses to global change was synchronic. Such approaches essentially test for local adaptation in the context of an environmental factor that varies spatially, but is predicted to change temporarily (the ‘space-for-time substitution’ approach, discussed by Merilä and Hendry 2013). This makes inferences on both the adaptive value of phenotypic divergence and the identification of the causal selection factor easier compared with allochronic data (Merilä and Hendry 2013). However, it is difficult to translate a spatial contrast into a temporal rate, both for the environmental parameter under study and for the rate of change in organismal phenotypes (Davis et al. 2005). A disadvantage common to all synchronic assessments is that they tell us something about past selection, while any inferences on rates of adaptation are difficult (Kinnison and Hendry 2001). In reciprocal transplants, the target environmental gradient, say temperature, often covaries with other features of the environment, often rendering inferences on the definitive selective agent inconclusive. One possible solution is the use of multiple, spatially independent gradients of the target factor when comparing populations as to decompose covarying effects (Kawecki and Ebert 2004; Oetjen and Reusch 2007). When population traits are compared in the laboratory, conditions are better controlled to unravel GxE interactions and causality of inferred selection regimes. The advantage of such an approach, the precise control of the environment is at the same time its disadvantage. As typically only one factor is manipulated, realistic upscaling to the multifactorial selection regime in the wild is difficult.

Among allochronic studies, I observed a dramatic lack of time series in the oceans that address phenotypic change in particular in the context of seasonality (fishes excluded), for example in photoperiodic cues for sporulation or flowering dates (macroalgae/seagrasses), in activity or migration patterns or in seasonal energy allocation patterns. It is also clear that even if initiated now, such time series would start to become instructive only much later. Some preserved specimen collections may be instructive to at least determine morphological shifts in, for example body size and form. An interesting alternative over monitoring programmes may be time series of revived genotypes obtained from resting stages stored in laminated sediments, for example from copepod resting eggs (Marcus et al. 1994). Such resurrection biology has been successfully applied to freshwater (Decaestecker et al. 2007) and marine plankton (Härnström et al. 2011) and allows for a direct comparison of genotype fitness as function of the presumed selection regime in common garden experiments using an allochronic approach.

As one direct approach to temporal phenotypic change, evolution experiments (Kawecki et al. 2012) are a largely underused method in marine evolutionary ecology in the context of global change, barring some notable exceptions (Kelly et al. 2012). Several invertebrate species have rapid population turnover in the order of weeks, such as small crustaceans, flatworms, appendicularians or rotatorians. Here, it would be very instructive to address evolutionary adaptation directly in replicated experiments with defined selection regimes. Interesting questions that could be addressed are the rate of environmental change, the importance of sexual reproduction and base population size, and the response to univariate and multivariate selection (Kawecki et al. 2012). The latter issue is particularly important, and several studies found pronounced interactive effects of the joint action of ocean warming and acidification on organismal performance, which taken together impose more organismal stress than each of the stressors alone (Pistevos et al. 2011). In some cases, adaptation to one stressor preadapts populations to another one, as shown for development time of sea urchins adapted to high temperature or low pH values (Foo et al. 2012). As many predicted stressors are highly correlated, such as temperature increase, pH drop and increases in oxygen deficiency (Boyd 2011), one useful strategy may be to design experiments that manipulate scenarios, rather than a decomposition of organismal effects to the single selection factors. This would be particularly cost and resource-effective if the question is whether or not particular key populations will persist via adaptation, rather than a causal determination of the precise selection regime (ER, see below).

One principal possibility to disentangle DNA-based evolutionary adaptation from plastic responses on one hand and of epigenetic from true genetic effects on the other is the direct assessment of (epi) changes at the molecular level (Reusch and Wood 2007; Danchin et al. 2011). However, this requires that we know the casual relationship between a genetic polymorphism or an epigenetic variant and the phenotype it produces in the first place. The rapid advances for the acquisition of genetic data even in nonmodel organisms, fuelled by next-generation sequencing technologies, have stimulated the rapidly growing field of ecological and environmental genomics that addresses the genetic basis of phenotypic change as a function of the environment (Feder and Mitchell-Olds 2003). Often and contrary to earlier enthusiasm (Reusch and Wood 2007), the way to a phenotype–genotype map turned out to be much harder than initially envisaged (Mackay et al. 2009; Travisano and Shaw 2013), and good examples that demonstrate causality are confined to a handful of cases among the fishes (DiMichele and Powers 1982; Colosimo et al. 2005). While the genome-wide study of polymorphisms is an interesting goal in and among itself, researchers should question themselves twice before embarking on large-scale acquisition of genetic/genomic data to unravel the genetic basis of global change related traits. If the research question is on evolutionary adaptation and the concomitant traits that confer increased fitness under environmental change, approaches at the level of phenotypic traits, their role for fitness and the underlying selection differentials and character correlations are more appropriate and resource-effective (see also Travisano and Shaw 2013).

A useful but underused strategy is certainly to apply combinations of approaches that mutually complement each other. Notable examples are studies that combine a breeding design along with exposure to the focal factor in either common garden experiments or via outplanting (Parsons 1997) or that combine assessments of narrow-sense heritabilities with selection experiments (Kelly et al. 2012). Such breeding designs also allow for an assessment of paternal and maternal nongenetic effects (as components of overall phenotypic plasticity) that turn out to be very important in marine systems for phenotypic buffering in the face of increasing stress (Donelson et al. 2011; Miller et al. 2012). Another successful example is short-term selection experiments, combined with the assessment of global changes in allelic composition of populations (Pespeni et al. 2013).

## Evolutionary projections

As longer term evolution experiments are often unfeasible in marine animals with complex life cycles or long generation times, one important novel direction is the combination of assessments of additive genetic variance with projective modelling of selection responses (Lynch and Lande 1993). The motivation for such approaches is rather an exploration of possible adaptive processes, rather than providing hard evidence for adaptive versus plastic changes, as discussed in Merilä and Hendry 2013 (this issue). Evolutionary projections have been applied in a few invertebrate species (Sunday et al. 2011; Kelly et al. 2013). For example, in the sea urchin *S. purpuratus*, the effects of ocean acidification on larval size (as surrogate for growth and later survival) were up to 50% smaller when accounting for adaptive evolution in a model considering measurements of additive genetic variance for size (as proxy for fitness) and predicting the rate at which a suboptimal phenotype returns to its optimal state by stabilizing selection. Note that approaches using *h*^2^ and selection differentials are a useful first step, but they have their inherent shortcomings. For example, due to trait correlations, the erosion of genetic variance under strong directional selection and fluctuating selection regimes, their predictions are often not very accurate (Merilä et al. 2001). To successfully project adaptive responses, it will be required to assess correlations among key traits important under global change (the G-matrix; Lynch and Walsh 1998). Trait correlations as a result of pleiotropy or genetic correlations may slow down evolutionary responses to climate change (Etterson and Shaw 2001), but in other cases, they can also enhance rates of adaptive evolution (Stanton et al. 2000).

## The evolution of reaction norms

In the published literature, almost all organisms came from coastal to near-shore habitats (Tables 2, 3). The somewhat paradoxical situation is that those organisms that are easily accessible and can be cultivated and raised under laboratory conditions are often ‘stress’ tolerators, already exposed to higher natural variation in temperature, oxygen deficiency and pH values compared with open-ocean areas (Silliman et al. 2005; Somero 2012) where the environment is more buffered (Reusch and Boyd 2013). Relevant environmental fluctuations are on a scale of hours to weeks hence most often ‘fine-grained’, that is, shorter than their generation time for many metazoan animals and plants. This variability is going to increase, for example by heat waves, upwelling of low pH/low oxygen waters or by extreme wind events and turbidity/light attenuation (Harley et al. 2006; Hoegh-Guldberg et al. 2007). None of the cited experimental designs directly addressed the capability of marine animals and plants to cope with enhanced environmental fluctuations. Theory predicts that organisms under fine-grained fluctuations generalist with respect to adaptive plasticity and tolerances will evolve, while those under constant conditions will be specialists with narrow tolerances (van Tienderen 1991; Scheiner 1993). As all environmental parameters in the ocean vary in space and time, a salient question is whether predicted changes at a locality will surpass present-day extremes. As an example, for ocean acidification, rates of change in pH levels are unprecedented for open-ocean habitats (Caldeira and Wickett 2003), yet, there are upwelling situations in which future levels are exceeded already now, which represent interesting and underexplored natural experiments. Note, however, that ocean acidification in hypoxic, CO_2_-enriched coastal systems will lead to peaks in pCO_2_ of 2000–4000 μatm within this century, thus greatly surpassing expected changes in the pelagic, open ocean (Feely et al. 2008; Melzner et al. 2013).

Conversely, genetically based adaptation to continually changing environments such as the open ocean may be more important in oceanic species, which would probably be realized by average trait evolution. Across the principal open ocean/coastal divide, a systematic study of the evolution of increased plasticity, respectively, phenotypic buffering in target populations/species is highly warranted. Testable hypotheses are that species/populations already possessing buffering/plasticity at the margins of their tolerances would adapt faster under the new extreme regime owing to genetic assimilation (Waddington 1959; Lande 2009). High phenotypic plasticity (both phenotypic buffering and plasticity *sensu stricto*) could hence be a precursor of mean trait changes. Alternatively, direct evolution of increasing plasticity, in the sense of steeper reaction norm slopes, is also possible and may be favoured by enhanced environmental variability predicted under global change (Thompson 1991; Chevin et al. 2013a,b). Thirdly, we have currently only a very poor understanding of costs associated with enhanced tolerances (Pigliucci 2005) that is prerequisite to predict the evolution of plasticity patterns and underlying reaction norm shapes. Thus, somewhat in contrast to the general theme of this review series, the study on how reaction norms and hence plasticity patterns evolve may guide a research programme on global change and evolution in the oceans (Thompson 1991; Pigliucci 2005; Chevin et al. 2013a,b).

## Conclusion – an evolutionary rescue perspective

Given the many examples cited in this review, it is almost trivial to find genetic differentiation between populations living in contrasting habitats for traits important under global change. Likewise, standing genetic variation for such traits seems to be abundant, at least in near-shore animal species and plants. In the absence of empirical time series, what we really need to know is how the potential for adaptation plays out, that is, whether or not populations at a locality will be rescued by evolution under increasing warming or acidification stress. Models exist that describe the rate of adaptation necessary to maintain positive population growth rates under directional change of the environment (Lynch and Lande 1993; Gomulkiewicz and Holt 1995). Such an ER approach for wild populations requires that we also have informed guesses about population demography processes, as the initial decline of maladaptive phenotypes subjects local populations to demographic stochasticity (Gomulkiewicz and Holt 1995) and lowers effective population sizes (Willi et al. 2006). Phenotypic plasticity needs to be integrated into ER approaches, as plastic/buffering responses may keep populations above a critical threshold until adaptive evolution has improved mean population fitness upon environmental change (Lande 2009). It was recently shown that plasticity interacts with the environmental sensitivity of a trait to selection, which describes the distance that the mean trait value is pushed away from the optimal value phenotypic value (Chevin et al. 2010). The beneficial effect of maintaining the trait closer to optimal values offsets the decelerated genetic selection response, hence, plasticity favoured ER (Chevin et al. 2010).

Evolutionary rescue can either be addressed by experimental evolution experiments (Bell and Gonzalez 2009) or its likelihood can be inferred from laboratory-based estimate of adaptive genetic variance along with field data on population sizes and projections of selection regimes. While such model predictions have inherent shortcomings (Merilä et al. 2001; Merilä and Hendry 2013), these approaches may be the only possibility for any educated guess for keystone animals and plants that are either long-lived and/or difficult to cultivate in the laboratory over longer time. Recent extensions of ER experiments have exposed artificially assembled trophic webs to environmental deterioration, clearly a very promising way to move forward that could be extended to entire marine planktonic food webs (Kovach-Orr and Fussmann 2013). Another fruitful extension is to include dispersal within a metapopulation context in which immigrating alleles may rescue local demes that otherwise would face extinction. So far, such experiments have only been conducted using laboratory model organisms such as yeast (Bell and Gonzalez 2011) and should be expanded to selected marine organisms within a gradient of population connectivity.

The main motivation for this review series was to confirm or refute evidence for phenotypic change as a result of (adaptive) evolution, based on genetic changes in wild populations. Owing to the lack of time series in marine populations other than abundance and distribution, I have largely explored the scattered evidence for the potential of adaptive evolution in the wild. Clearly, it will be impossible to study the entire taxonomic diversity of marine animals and plants, but a more systematic study of major life-history types, population sizes, habitat types (coastal versus open ocean) and migration capacities is highly warranted.
